# The French Emergency National Survey: A description of emergency departments and patients in France

**DOI:** 10.1371/journal.pone.0198474

**Published:** 2018-06-14

**Authors:** Diane Naouri, Carlos El Khoury, Christophe Vincent-Cassy, Albert Vuagnat, Jeannot Schmidt, Youri Yordanov, for the French Society of Emergency Medicine Evaluation and Quality Committee

**Affiliations:** 1 Sorbonne Universités, UPMC Paris Univ-06, Paris, France; 2 Emergency Département, Hôpital Saint-Antoine, Assistance Publique-Hôpitaux de Paris, Paris, France; 3 Emergency Department and RESCUe Network, Lucien Hussel Hospital, Vienne, France; 4 Univ. Lyon, Claude Bernard Lyon 1 University, HESPER EA 7425, Lyon, France; 5 Emergency Département, Hôpital Kremlin Bicêtre, Assistance Publique-Hôpitaux de Paris, Le Kremlin-Bicêtre, France; 6 Directorate for Research, Studies, Evaluation and Statistics of the French Health and Social Affairs Ministry, Paris, France; 7 Emergency Department, Clermont-Ferrand University Hospital, Clermont-Ferrand, France; 8 EA 4679, Université Clermont Auvergne, Clermont-Ferrand, France; 9 INSERM, U1153, Paris, France - Centre d’Épidémiologie Clinique, Hôpital Hôtel Dieu, Assistance Publique–Hôpitaux de Paris (APHP), Paris, France; King Abdullah International Medical Research Center, SAUDI ARABIA

## Abstract

**Introduction:**

Some major changes have occurred in emergency department (ED) organization since the early 2000s, such as the establishment of triage nurses and short-track systems. The objectives of this study were to describe the characteristics of French EDs organization and users, based on a nationwide cross-sectional survey.

**Methods:**

The French Emergency Survey was a nationwide cross-sectional survey. All patients presenting to all EDs during a 24-hr period of June 2013 were included. Data collection concerned ED characteristics as well as patient characteristics.

**Results:**

Among the 736 EDs in France, 734 were surveyed. Triage nurses and short-track systems were respectively implemented in 73% and 41% of general EDs. The median proportion of patients aged > 75 years was 14% and median hospitalisation rate was 20%. During the study period, 48,711 patients presented to one of the 734 EDs surveyed. Among them, 7% reported having no supplementary health or universal coverage (for people with lower incomes). Overall, 50% of adult patients had been seen by the triage nurse in less than 5 minutes, 74% had a time to first medical contact shorter than one hour and 55% had an ED length of stay shorter than 3 hours.

**Conclusion:**

The French Emergency Survey is the first study to provide data on almost all EDs in France. It underlines how ED organization has been redesigned to face the increase in the annual census. French EDs appear to have a particular role for vulnerable people: age-related vulnerability and socio-economic vulnerability with an over-representation of patients without complementary health coverage.

## Introduction

The main aim of emergency medicine (EM) is prevention, diagnosis, treatment and orientation for patients with a wide range of un-anticipated illnesses or injuries [1–4]. Emergency physicians (EPs) are often the first contact with the health care system for patients presenting an emergency medical condition, regardless of gender, age, insurance status and time of day [2–5]. In the French system, different outpatient and hospital providers collaborate to deliver the most appropriate level of care in emergency situations, mainly EPs and general practitioners (GPs) [6,7]. Several “out-of-hours” services exist to give patients access to a GP at night, on weekdays and all day on weekends or public holidays. A hospital-based department, the *Service d’Aide Médicale Urgente* (SAMU), offers a 24/7 telephone medical advice service, and whenever necessary, can send mobile intensive care units (MICUs), with EPs, in the pre-hospital field. Ultimately, patients seeking urgent care can visit the emergency department (ED) of any hospital they choose, at their own initiative or upon referral from one of the previous providers [6,7]. In the last 15 years, ED visits have been steadily increasing worldwide [5,8–12]. French EDs share this situation with a 45% increase in ED visits during this period of time [7,8]. EDs became a common hospital admission route, as even if scheduled admissions appear to be stable, the number of patients admitted to the hospital via the ED (i.e unscheduled admissions) increased from 3.6 to 4.7 million/year from 2004 to 2011, thus reflecting that unscheduled admissions are becoming a larger part in total admissions [13]. The increase of non-urgent [14] and repeated visits [15–18] contribute of the increase in the number of total ED visit and ED overcrowding which is known to be associated with an increased morbi-mortality [19,20], increased frequency of medical errors [21] and reduced satisfaction of patients [22].

Almost 15 years ago, a major reorganization of emergency medicine was set in motion by the emergency care actors and the French government [23,24]. Several measures, as triage nurses or ED short-track systems, were introduced. The triage system consists of basic clinical assessment that aims to determine in how much time the patient should be seen and the amount of resources to be used. In France, there is no homogeneity in the use of triage scores but the most frequently used is the FRENCH triage scale [25]. Short-track systems set apart patients with non-urgent complaints and possible rapid discharge in a dedicated area, limiting fragmentation of care [26]. They both aim to decrease waiting times, ED length of stay, reduce ED overcrowding and increase patient and staff satisfaction [26]. However, we lack data to comprehensively describe emergency care after these major changes. This study aimed to examine and describe French ED organization and ED patients, based on a 1-day nationwide cross-sectional survey.

## Methods

### Study design and selection of participants

The **F**rench **E**mergency **S**urvey (FES) was a nationwide cross-sectional survey with a two-level design aiming to describe hospital-based emergency care in France through ED organization and ED patients. It was developed by the Directorate for Research, Studies, Evaluation and Statistics of the French Health and Social Affairs Ministry (DREES) with the help of the French Society of Emergency Medicine (SFMU), and the SAMU French ED association (SUdF).

The study took place on June 11, 2013, in all emergency departments in France (as defined by the decree 2006–577 [24]). Any patients presenting to an ED in France during the study period (24-hr, June 11^th^, 2013 from 8:00 am to June 12^th^, 2013 8:00 am) were included. June 11 was a weekday (Tuesday) and the month of June has a high rate of visits [27], which allowed inclusion of a large number of patients that could be representative of ED patients.

### Survey development

The research tool was developed by the study steering committee based on a previous survey of a sample of EDs [28], a literature review and a qualitative study as previously described [28]. Briefly, the steering committee searched MEDLINE via PubMed, RefDoc, ameli.fr, and grey literature (including scientific society websites and scientific meetings proceedings) for all articles and reports of emergency medicine and ED organization in France. In total, 36 studies and reports were identified. Then a qualitative study was conducted among the main institutional actors involved in France’s emergency care. All interviews were performed from December 23, 2011 to March 8, 2012. A total of 33 institutions were approached and their representatives interviewed. These institutions were the French Health and Social Affairs Ministry, Regional Health Agencies, French National Health Insurance Fund for Salaried Employees, Emergencies Regional Observatories (ORU), National Medical Council, federations of health care facilities, university and general hospitals, EP and GP unions and nursing home physicians. Patient representatives were also interviewed. After that preliminary phase, the research tool was tested with 23 EDs in February 2013 and modified according to their feedback.

### Survey description

A two-level design was used for the survey. The first part was an ED-centered questionnaire, aimed at describing the surveyed hospital and ED. It was to be completed once by each ED administrator. The second part was the patient questionnaire, which was to be completed by the emergency physician for each patient who presented to any of the surveyed EDs during the study period.

### ED questionnaire

The ED questionnaire comprised 105 questions organised as follows: 1) department identification (e.g., name, localization, adult or paediatric ED); 2) local organization of emergency care (e.g., existence of a short-track system, existence of direct access to specialized care [cardiology, neurology, gynaecology, geriatrics and ophthalmology]; 3) human resources (e.g., number of doctors, residents, nurses and other healthcare workers); 4) department organization (e.g., presence of a triage nurse, social workers, paediatrician, psychiatrists and/or inpatient geriatric consultation); 5) collaboration with the other hospital departments (e.g., resuscitation procedures, surgical, medical and geriatric consult, CT and MRI accessibility and for how many hours per day, availability of hospital rooms, presence of a bed manager, existence of a local major incidents management plan [as recommended by the Circular No. DHOS/CGR/2006/401 of September 14, 2006 [29]]); and 6) emergency attendance the day of the study.

### Patient questionnaire

For every patient who presented to a participating ED on that day, the patient section of the questionnaire was completed by the patient or the accompanying person under the supervision of the EP. The patient section included up to 123 questions. Sociodemographic questions included age, gender, level of education (none / less than high school degree / high school degree / more than high school degree), employment status as defined by the French national institute for statistical and economic studies (active [employed / unemployed] / inactive [retired / student / other inactive]), health care coverage (none / public health insurance / state medical assistance) and complementary health insurance coverage [none / private / universal complementary health care coverage (CMU-C)]. In France, a large part of the population enrols in a private complementary health insurance to cover co-payments required by public health insurance. People with less than an income threshold can benefit from a free complementary health insurance called the CMU-C. When the patient was a child, the sociodemographics reported were those of the accompanying family member. All patients were also asked about 1) the mode of arrival; 2) the genesis of the emergency consult (prior care procedures undertaken before the ED visit, self-reported reasons for the visit [isolated medical reason, difficulties in access to care, need for institutionalization etc.]); and 3) usual use of healthcare system and existence of a GP referent. Moreover, each step of care provided in the ED was recorded by the physician: initial complaint; final diagnosis; waiting times; biological, radiological or therapeutic interventions; and output mode (discharge, hospitalization etc.). Physicians were also asked to assess the adequacy of the emergency consult for each patient. Initial complaints were classified by using the SFMU thesaurus and the final diagnoses were matched according to their International Classification of Diseases (ICD-10) codes.

### Data collection

One study referent in each ED transmitted data from completed questionnaires to a dedicated secure website. All data were anonymous. An external provider helped with website conception and data collection. All steps of data collection were supervised by the DREES. Reminders were sent to non-responding EDs, both through the external service provider and the SFMU.

### Data quality

The DREES verified the completeness of the database. Internal consistency was, first, controlled by focusing on 3 major variables: age, gender and radiological prescriptions so as to detect discrepancies between age and type of ED (e.g., adult patients seen in a pediatric ED), gender and pathologies (e.g., male with a strictly female diagnosis) and type of radiological exam and type of ED (e.g., unconventional imaging procedure [MRI or tomodensitometry] in a structure that does not have such imaging available). For age and gender, less than 0.001% discrepancy was noted. For radiological exams, a 2% discrepancy was noted. This finding probably also reflects particular organizations, for example a partnership with a nearby imaging center. Then, data quality was assessed by comparing data from the FES and the Oscour^®^ network. The Oscour^®^ network was established in 2004 with support of the General Directorate of Health Care Provision, the SFMU and regional partners including the ORU. In 2013, 414 emergency structures participated in the monitoring network, covering nearly 65% of ED visits in France [27]. Data from EDs involved in the Oscour^®^ network are routinely obtained from patient medical records and the emergency passage summary, including sociodemographic, administrative and medical data. The Oscour^®^ network and FES data were consistent for ED visits recorded on June 11, 2013.

### Ethics

This study was declared to be of public interest by the CNIS (Conseil National d’Information Statistique) and was integrated into the public statistical program (Visa no. 2013X080SA and publication in the official Journal of French Republic September 17, 2013). It was also approved by the CNIL (Commission Nationale de l’Informatique et des Libertés, French law no. 78–17) (identification no. 1663413). According to French law, written informed consent was not required for this type of study. Patients were informed by staff and a short handout and posters were in the waiting area; 0.3% refused to participate.

### Statistical analysis

Descriptive analyses were performed. They included the characteristics of the EDs and then the characteristics of ED users. EDs users were classified in two ways: first according to the type of ED visited, then according to their age. Continuous variables, which were non-normally distributed, are reported as median (Q1-Q3). Categorical variables are reported as numbers (%). All statistical analyses were performed with SAS (SAS/STAT Package 2002–2003, SAS Institute Inc., Cary, NC, USA).

## Results

### Characteristics of all emergency departments

Among the 736 surveyed EDs, 734 (99.7%) provided data. Two types of ED were identified, general EDs (86%, n = 629) and EDs receiving only children; i.e., pediatric EDs (14%, n = 105). Among all EDs, 99 (13%) were in public academic hospitals, 472 (64%) public non academic hospitals, 45 (6%) not-for-profit private hospitals and 118 (16%) for-profit private hospitals. These characteristics are reported in Table 1.

**Table 1:** Characteristics of all emergency department.

	General ED	Pediatric ED	TOTAL
**N (%)**	629 (86)	105 (14)	734
**Type of hospital N (%)**			
Public Academic Hospitals	65 (10)	34 (32)	99 (13)
Public Non Academic Hospitals	405 (64)	67 (63)	472 (64)
Not-For-Profit Private Hospitals	41 (7)	4 (4)	45 (6)
For-Profit Private Hospitals	118 (19)	0	118 (16)
**Annual census median (Q1-Q3)**	23,177 (15,330–33,580)	16,790 (10,585–27,375)	22,265 (14,600–32,850)
≤ 15,000	150 (24)	47 (45)	197 (27)
15,001–30,000	267 (42)	43 (41)	310 (42)
30,001–45,000	127 (20)	8 (8)	135 (18)
> 45,000	84 (13)	7 (7)	91 (12)

The vast majority (85%, n = 536) of general EDs received only adult patients (n = 536). Annual census was 23,177 (Q1-Q3: 15,330–33,580). Less than 35% of EDs had more than 30,000 yearly visits, but more than 75% of these EDs were in public university hospitals. All characteristics are reported in Table 2. A triage nurse was available in 475 EDs (73%). In 39% of the departments (n = 245) availability was 24 hr/day (Table 3). Among hospitals with more than 45,000 yearly visits, 100% had a triage nurse and 81% had 24hr/day availability. Differences related to the type of hospital or annual census are reported respectively in Tables 2 and 3. Overall, 50% of patients had been seen by the triage nurse less than 5 minutes after arrival. A short-track system (which corresponds to the area dedicated for patients with non-urgent complaints and possible rapid discharge) was available in 255 EDs (41%). Differences related to type of hospital or annual census are reported respectively in Tables 2 and 3. About half the hospitals had a inpatient geriatric consultation team, mostly in public academic hospitals (82%; n = 53) and more rarely in for-profit private hospitals (10%; n = 12). A CT scanner was available in 576 EDs (92%) among which 87% (n = 550) had 24hr/day accessibility (Table 2). Median geriatric (> 75 years), pediatric and trauma patient rates were 14% (Q1-Q3:9–19), 17% (Q1-Q3:10–25) and 35% (Q1-Q3:28–45), respectively (Table 2). Median hospitalisation rate was 20% (Q1-Q3:14–27). Hospitalisation dashboard (i.e bed rooms availability in the hospital) was available in 91% of EDs (n = 574) with a dedicated staff in 24% of EDs (n = 152).

**Table 2:** Characteristics of general emergency departments.

	Public Academic Hospitals	Public Non Academic Hospitals	Not-For-Profit Private Hospitals	For-Profit Private Hospitals	TOTAL
**N (%)**	65 (10)	405 (64)	41 (7)	118 (19)	629
**Annual census median (Q1-Q3)**	43,070 (30,660–48,910)	23,360 (14,600–33,215)	21,535 (14,235–29200)	20,440 (15,695–25,915)	23,177 (15,330–33,580)
≤ 15,000	4 (6)	107 (26)	14 (34)	25 (21)	150 (24)
15,001–30,000	12 (18)	161 (40)	18 (44)	76 (64)	267 (42)
30,001–45,000	24 (37)	82 (20)	6 (15)	15 (13)	127 (20)
> 45,000	25 (38)	55 (14)	3 (7)	1 (1)	84 (13)
**Technical ressources N (%)**					
Tomodensitometry	65 (100)	355 (88)	40 (98)	116 (98)	576 (92)
*Whose 24h/24*	*63 (97)*	*344 (85)*	*38 (93)*	*105 (89)*	*550 (87)*
MRI	63 (97)	223 (55)	26 (63)	91 (77)	403 (64)
*Whose 24h/24*	*39 (60)*	*67 (17)*	*13 (32)*	*49 (42)*	*168 (27)*
**Short-track system**^a^ **N (%)**	32 (49)	160 (40)	19 (46)	44 (37)	255 (41)
**Geriatric patients rate median (Q1-Q3)**^b^	16 (12–19)	15 (11–20)	10 (7–17)	9 (5–14)	14 (9–19)
**Geriatrie mobil unit N (%)**	53 (82)	228 (56)	12 (29)	12 (10)	305 (48)
**Pediatric patients rate median (Q1-Q3)**^c^	0 (0–1)	20 (13–26)	14 (4–22)	20 (11–26)	17 (10–25)
**Pediatricien in ED N (%)**	1 (2)	56 (14)	1 (2)	8 (7)	66 (10)
**Traumatic patients rate median (Q1-Q3)**^d^	25 (18–32)	35 (29–43)	40 (32–47)	43 (32–52)	35 (28–45)
**Hospitalisation rate median (Q1-Q3)**	24 (19–31)	22 (16–28)	16 (13–20)	12 (7–19)	20 (14–27)

^a^ED short-track systems correspond to dedicated area and physician to care patients with lowest level of urgency

^b^Geriatric patient is defined as patient aged more than 75 years

^c^Pediatric patient is defined as patient aged less than 15 years

^d^Traumatic patient is defined as patient with traumatic injury complaint

**Table 3:** Characteristics of triage organization depending on the number of ED visits per year.

	Annual census				
	≤ 15,000	15,001–30,000	30,001–45,000	≥ 45,000	TOTAL
**Triage nurse—N (%)**	55 (37)	196 (73)	122 (96)	84 (100)	458 (73)
**Triage nurse 24h/24—N (%)**	28 (19)	72 (27)	76 (60)	68 (81)	245 (39)
**Percentage of nurses trained for triage median (Q1–Q3)**	21 (10–50)	50 (16–70)	51 (20–76)	62 (40–78)	50 (16–72)

### Characteristics of pediatric EDs

For the 105 pediatric EDs, 34 (32%) were in public academic hospitals, 67 (63%) public non academic hospitals, and 4 (4%) not-for-profit private hospitals, none in for-profit private hospitals (Table 1). Annual census was 16,790 (Q1-Q3:10,585–27,375), and more than 85% of EDs had fewer than 30,000 visits per year.

### Characteristics of ED users and care

Among the 52,018 expected questionnaires (based on the number of ED visits to each ED during the study period, as described in the ED section of the survey) 48,711 patient questionnaires were collected (94%). They were completed by the accompanying person/family member (in most cases, the accompanying person was the parent) in 25% of cases. For 2% of patients, data collection was impossible because the patient was not able to communicate and no accompanying person was available.

### Users older than 15 years (Table 4)

**Table 4:** Characteristics of users older than 15 years.

	Total (N = 34,925)
**Age N (%)**	
15–25 years	6285 (18)
26–35 years	6045 (17)
36–45 years	5198 (15)
46–55 years	4627 (13)
56–65 years	3891 (11)
66–75 years	2899 (8)
76–85 years	3372 (10)
> 85 years	2608 (7)
**Gender N (%)**	
Male	17882 (51)
Female	16596 (48)
Missing data	447 (1)
**Complementary health coverage N (%)**	
None	2326 (7)
Universal complementary health coverage	2572 (7)
Private	24007 (69)
Missing data	6020 (17)
**Health insurance N (%)**	
None	532 (2)
State medical assistance (AME)	276 (1)
Public health insurance	29681 (85)
Missing data	4436 (12)
**Employment status N (%)**	
Employed	12868 (37)
Unemployed	2465 (7)
Retired	8372 (24)
Student	2103 (6)
Other Inactive	1823 (5)
Missing data	7294 (21)
**Level of education N (%)**	
No graduate	7072 (20)
Less than high school degree	8776 (25)
High school degree	5043 (14)
More than high school degree	5212 (15)
Missing data	8822 (25)
**Living conditions N (%)**	
Home, not alone	10524 (30)
Home, alone	19120 (55)
Institution	1173 (3)
Homeless	200 (1)
Hotel	49 (< 1)
Missing data	3859 (11)
**Mode of arrival N (%)**	
By his own means	14714 (42)
By car with another person	6809 (19)
Taxi or private ambulance	5082 (15)
Firefighters	4683 (13)
MICUs	574 (2)
Security forces	438 (1)
Missing data	2625 (8)
**Provenance N (%)**	
Home	22472 (64)
Public place	6512 (19)
Another hospital	577 (2)
Institution for disabled persons	940 (3)
Primary care center	153 (< 1)
Missing data	4271 (12)
**ED visit in the last 24 hours N (%)**	
Yes in the same ED	1029 (3)
Yes in another ED	316 (1)
No	29972 (86)
Missing data	3608 (10)
**ED visit in the last 7 days N (%)**	
Yes in the same ED	1500 (4)
Yes in another ED	426 (1)
No	29367 (84)
Missing data	3632 (10)
**Onset of complaint N (%)**	
The day of ED visit	16690 (48)
The day before ED visit	4572 (13)
More than one day before ED visit	10041 (29)
Missing data	3222 (9)
**Time of ED arrival N (%)**	
8h-11h59	8818 (25)
12h-15h59	8671 (25)
16h-19h59	8293 (24)
20h-23h59	5702 (16)
00h-3h59	1972 (6)
4h-8h	1469 (4)
**Output mode N (%)**	
Return home	24893 (71)
Hospitalization at home (HAD)	200 (1)
Hospitalization	7272 (21)
Hospitalization in another hospital	1001 (3)
Return to elderly institution (EHPAD)	412 (1)
Death	38 (< 1)
Exit against medical advice	223 (1)
Redirected to a primary care center	51 (< 1)
Gone without waiting	785 (2)
Missing data	50 (< 1)

**Fig 1:**
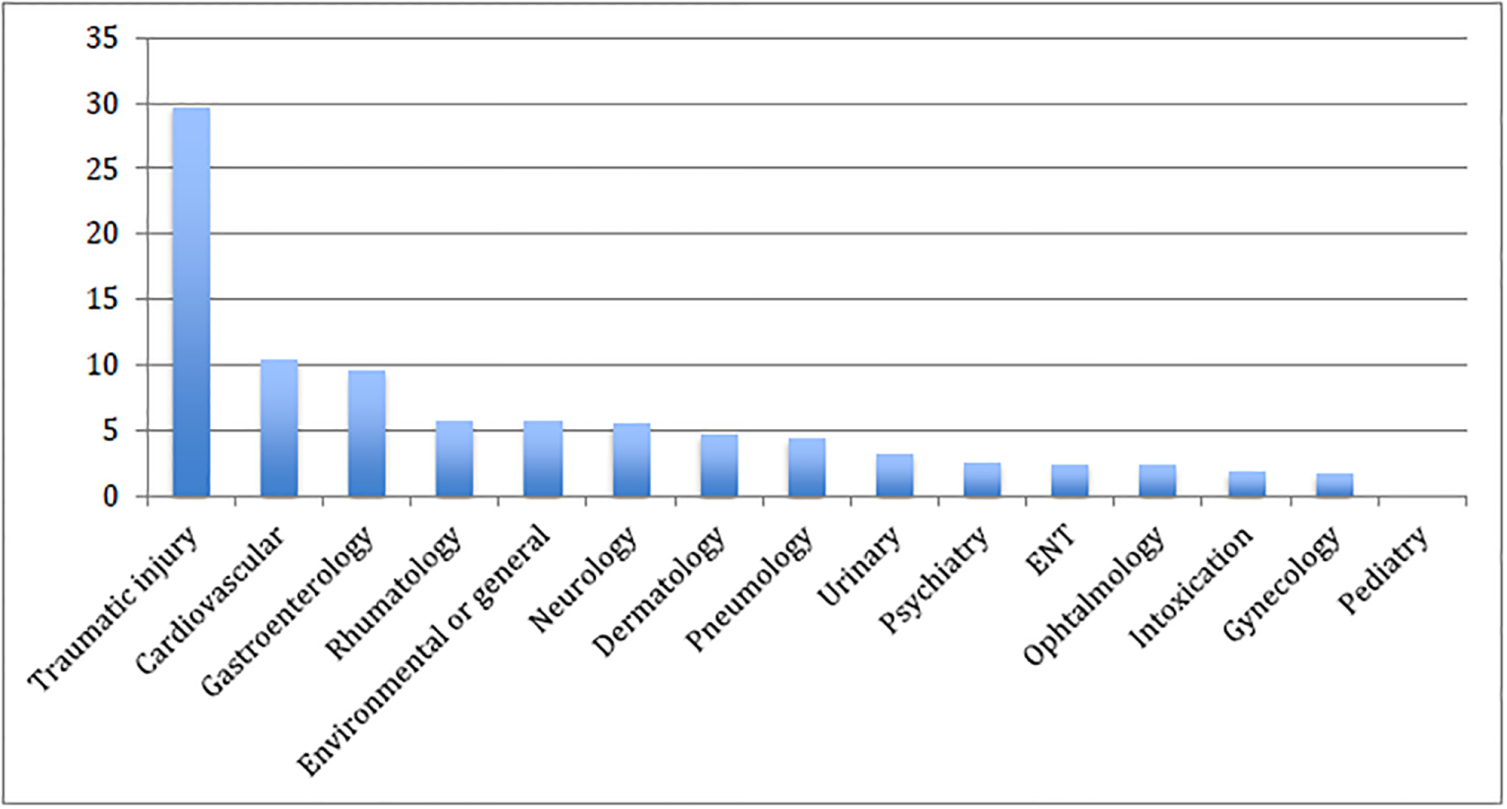
Type of chief complaint of user older than 15 years.

Among the 34,925 patients, older people (> 75 years) represented 17% of patients (n = 5980). Overall, 69% (n = 24,007) had complementary private insurance coverage, and 7% (n = 2572) had supplementary universal health coverage (CMU-C). About 45% (n = 15,848) hadn’t completed high school and 37% (n = 12,868) of all patients were employed. About 75% of patients self-referred to the ED (n = 26,284). Approximatively 48% (n = 16,690) sought care for complaints that had been present for less than 24 hrs. About 74% (n = 25,782) had consulted during usual open hours of outpatient care (8 am to 8 pm). The chief complaints of consultation are presented in Fig 1. The most frequent were traumatic injuries (30%), cardiovascular symptoms (10%) and gastroenterological symptoms (10%). Decreased general condition and loss of autonomy represented 2% (n = 557) and less than 1% (n = 58) of chief complaints, respectively. The most frequent cardiovascular and gastroenterological symptoms were chest pain (44%) and abdominal pain (73%). Overall, 25% of patients (n = 8597) had been seen in a short track system. Time to first medical contact after triage was shorter than one hour for 74% of patients (n = 25,883). ED length of stay (LOS) was shorter than 3 hours for 55% of patients (n = 19,165).

### Users younger than 15 years (Table 5)

**Table 5:** Characteristics of users younger than 15 years.

	Total (N = 12,896)
**Age N (%)**	
< 18 month	2081 (16)
18 month—5 years	3346 (26)
6–10 years	3272 (25)
11–15 years	4197 (33)
**Gender N (%)**	
Male	7074 (55)
Female	5601 (43)
Missing data	221 (2)
**Mode of arrival N (%)**	
By parents	9959 (77)
By car by another person than parents	1317 (10)
Taxi or private ambulance	305 (2)
Firefighters	702 (5)
MICUs	63 (<1)
Security forces	4 (<1)
Missing data	546 (4)
**Provenance N (%)**	
Home	9144 (71)
Public place	2527 (20)
Hospital	134 (1)
Institution for disabled person	95 (<1)
Primary care center	69 (<1)
Missing data	1017 (8)
**ED visit in the last 24 hours N (%)**	
Yes in the same ED	470 (4)
Yes in another ED	135 (1)
No	11624 (90)
Missing data	667 (5)
**ED visit in the last 7 days N (%)**	
Yes in the same ED	639 (5)
Yes in another ED	165 (1)
No	11416 (89)
Missing data	676 (5)
**Onset of complaint N (%)**	
The same day of ED visit	7299 (57)
One day before ED visit	2036 (16)
More than one day before ED visit	2916 (23)
Missing data	645 (5)
**Time arrival N (%)**	
8h-11h59	2692 (21)
12h-15h59	3073 (24)
16h-19h59	3819 (30)
20h-23h59	2519 (20)
00h-3h59	530 (4)
4h-8h	263 (2)
**Output mode N (%)**	
Return to home	11270 (87)
Hospitalization at home (HAD)	82 (<1)
Hospitalization	1201 (9)
Hospitalization in another hospital	116 (<1)
Return to institution	8 (<1)
Death	1 (<1)
Exit against medical advice	13 (<1)
Redirected to a primary care center	26 (<1)
Gone without waiting	164 (1)
Missing data	15 (<1)

**Fig 2:**
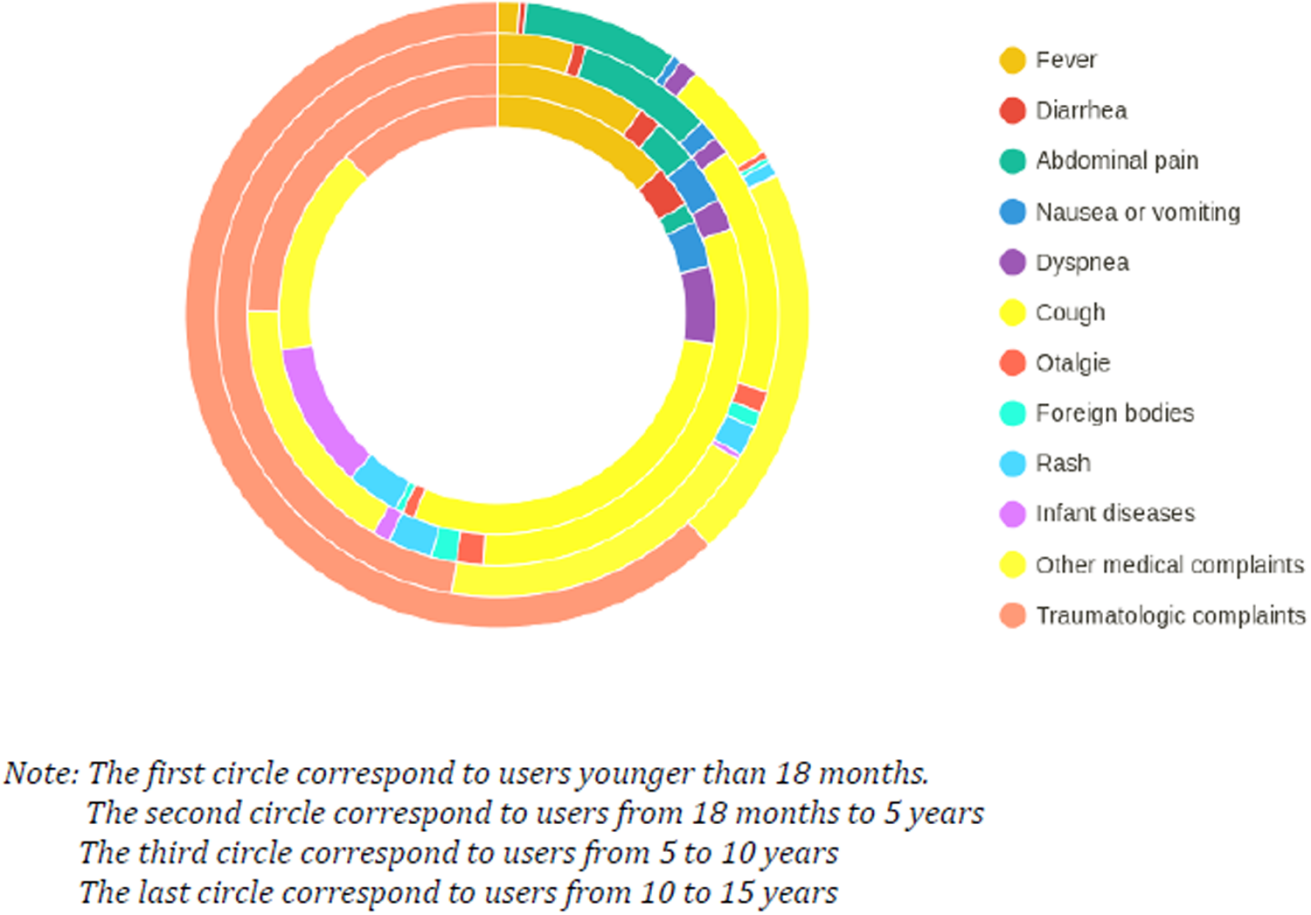
Type of chief complaint of user younger than 15 years, by age.

Among the 12,896 patients younger than 15 years, about 40% (n = 5432) had consulted in a paediatric ED. Approximately 57% (n = 7299) sought care for problems that had been present for less than 24 hrs. About 74% (n = 9584) had consulted during usual open hours of outpatient care (8 am to 8 pm). Chief complaints are represented per age in Fig 2.

## Discussion

To the best of our knowledge, the French Emergency Survey is the first study that provides data aimed to portray the characteristics of all EDs in a European Country with such exhaustivity [30]. A previous study performed in 2002 among a sample of 150 French EDs (and considered as representative of all French EDs) reported an annual census of 23,000 [31]. In our study, this number was 26,500 corresponding to an increase of 15% in 10 years.

The triage nurse concept was introduced in 1991 in French EDs [32]. The decree No 2006–577 regarding ED organization [24] recommends that when the activity of the emergency structure allows it, the team should also include a triage nurse. In 2013, the SFMU published triage recommendations [33] calling for a triage nurse in all EDs with more than 5 patients per hour (corresponding to 43,800 ED visits per year). In our study, every ED with more than 45,000 ED visits per year had a triage nurse. Numerous international studies have been published about the use of triage scales [34,35]. But few data are available regarding the rate of triage implementation in developed countries. A study reported in 2011 that 97% of Swedish EDs used triage scales compared to 50% in the early 2000’s [36]. To mitigate overcrowding, triage, as well as other interventions that aim at improving patient flow, has been extensively evaluated. Short tracks have been shown to be effective in reducing low priority patients waiting time and length of stay, without negatively affecting the times of patients with higher priority [26,37–41]. In our study, 41% of general EDs had a short-track system which concerned 25% of adult patients.

Among patients older than 15 years, 7% had universal supplementary health coverage (CMU-C) and 7% no supplementary health insurance coverage. In 2010, according to the French Institute for Research and Information in Health Economics [42–44], 94.7% of the French population had supplementary health insurance coverage: 89.0% private supplementary health insurance coverage and 5.7% CMU-C coverage. Only 4.2% had no supplementary health insurance coverage. Our results show that ED patients were less likely to have a supplementary health insurance or CMU-C coverage, as compared to the overall French population. Numerous studies have demonstrated that no or poor supplementary health insurance coverage is associated with both difficulties in health care access and frequency of ED visits [45–48]. Thus, over-representation of people with no supplementary health insurance coverage or CMU-c might reflect the social vulnerability of this population.

Finally, median hospitalization rate was 20%. We found approximately the same hospitalization rate as the previous study performed in 2003 [31]. As in 2003, the median hospitalization rate was higher in public hospitals than private hospitals [31].

### Study limitations

The main limitation is that data regarding patients were recorded in real time (i.e., during patient care), which can suggest reporting errors. Second, this study has limitations that are common to this type of study design (declarative surveys) such as reporting bias (including social desirability bias) and representativeness of the sample. This is especially true if the patient-questionnaire was completed with the assistance of a proxy (in our study 25% of patients). But it is important to note that in most cases, the proxy was a family member. Thus, we can assume that the proxy is sufficiently close to the individual to correctly answer, particularly concerning socio-demographic characteristics. In addition, the design of the survey allowed for responses to the ED questionnaire provided by an ED administrator, which reduced the risk of missing data and reporting bias. Concerning representativeness of the sample, as 99.7% of French EDs provided data with a response rate of 94% for patient questionnaire, we can assume that our study sample is representative of French EDs and patients.

And finally, data referred to French EDs, which raises the question of the generalizability of the results. In 2002, the French Health and Social Affairs Ministry compared the organization of emergency systems in 10 European countries [49] and reported that major differences existed for pre-hospital care but that EDs were similar.

## Conclusion

The **F**rench **E**mergency **S**urvey provides data regarding French EDs and their organization. It also describes precisely ED patients’ characteristics, details of ED care and length of stay. In addition, it underlines how ED organization has been redesigned to face the increase in the number of ED visits, such as the establishment of a triage nurse or of a fast-track process. Moreover, French EDs appear to have a particular role for vulnerable people, age-related vulnerability as one in five patients are more than 75 years old, but also socio-economic vulnerability, with an over-representation of patients with no complementary health coverage. These major considerations might guide future studies on the subject of ED organization and care.

## Supporting information

S1 File Appendix glossary. Glossary of abbreviations used.
(DOCX)
