# Supplementary material for: The French Emergency National Survey: A description of emergency departments and patients in France
Source: PLoS One. 2018 Jun 14;13(6):e0198474. doi: 10.1371/journal.pone.0198474 (PMC6002101; doi:10.1371/journal.pone.0198474)
Supplement: S1 File — Glossary of abbreviations used. (DOCX) [file pone.0198474.s001.docx]

**S1 File. Appendix – Glossary**

CMUc Universal complementary health care coverage

CNIS National Council for Statistical Information

CNIL French National Agency regulating Data Protection

CT Computerized tomography

DREES Directorate for Research, Studies, Evaluation and Statistics of the French Health and Social Affairs Ministry

ED Emergency department

EHPAD Elderly institution

EP Emergency physician

FES French Emergency Survey

HAD Hospitalization at home

MICUs Mobile intensive care units

MRI Magnetic resonance imaging

ORU Emergencies Regional Observatories

SFMU French Society of Emergency Medicine
